# Surgical Management of Choanal Atresia With Intranasal Hegar’s Dilator and Transnasal Endoscopic Excision: A 20-year Retrospective Comparative Study

**DOI:** 10.7759/cureus.8060

**Published:** 2020-05-11

**Authors:** Mohammad A Alshareef, Abdullah S Assalem, Fatimah Alzubaidi, Basem Damanhouri, Tariq A Al-Aidarous

**Affiliations:** 1 Otolaryngology - Head and Neck Surgery, King Abdulaziz Medical City, Jeddah, SAU; 2 Otolaryngology - Head and Neck Surgery, Armed Forces Hospital, Ministry of Defense, Taif, SAU; 3 Head, Neck and Skull Base Center, King Abdullah Medical City, Makkah, SAU; 4 Otolaryngology - Head and Neck Surgery, Al-Noor Specialist Hospital, Makkah, SAU

**Keywords:** choanal atresia, surgery, intranasal, hegar’s dilator, transnasal, endoscopy, excision

## Abstract

Background

Although choanal atresia (CA) was first described 250 years ago, its description and understanding remain incomplete, as is the distinction between unilateral and bilateral CA. Among the surgical techniques introduced to manage this condition are intranasal Hegar’s dilator (IHD) and transnasal endoscopic excision (TNEE).

Objectives

This study retrospectively evaluated the outcomes and effectiveness of IHD and TNEE in the treatment of patients with CA, including differences in the incidence of re-stenosis with these techniques.

Methods

Patients diagnosed with CA who underwent surgical interventions in the Otolaryngology Department of Al-Noor Specialist Hospital, Makkah, Saudi Arabia, between 1997 and 2017 were analyzed. Postoperative outcomes including re-stenosis rates were compared in patients who underwent IHD and TNEE. Factors associated with patient outcomes were analyzed, including ages at diagnosis and surgery, nationality, gender, type of atresia (unilateral/bilateral and bony/membranous/mixed), surgical intervention (IHD or TNEE), and re-stenosis and need for revision surgery after IHD and TNEE.

Results

A total of 30 patients were diagnosed with CA, including 21 (70%) girls and 9 (30%) boys. Of them, 18 (60%) patients were diagnosed at younger than one month of age, 28 (93%) were Saudi nationals, and 20 (67.70%) were aged younger than three months at the time of surgery. Of these 30 patients, 17 (56.70%, all Saudi nationals) underwent IHD, and 13 (43.30%), including 15 Saudi nationals, underwent TNEE. The 17 patients who underwent IHD included 13 (76.50%) girls and 4 (23.50%) boys, whereas the 13 patients who underwent TNEE included 8 (61.50%) girls and 5 (38.50%) boys. Fifteen patients (50%) had mixed-type CA, nine (30%) had bony-type CA, and six (20%) had membranous-type CA. Twenty-six (86.67%) patients underwent primary surgery, whereas four (13.33%) underwent revision surgery; of the latter, three (75%) had undergone primary IHD, and one had undergone primary TNEE. Only one (3.33%) patient experienced re-stenosis after revision surgery, which consisted of IHD. Twelve patients (40%) underwent stenting, with one developing re-stenosis. The relationships between surgical approach and re-stenosis after primary and secondary surgery were not statistically significant.

Conclusion

The outcomes in patients with CA treated with IHD and TNEE are comparable. Rates of re-stenosis and need for revision surgery do not differ significantly in patients treated with these surgical approaches.

## Introduction

Choanal atresia (CA), a rare congenital disfigurement of the nasal cavity first described in the mid-18th century, is generally characterized by the absolute obliteration of the posterior nasal choanae, which interferes with respiratory airflow from the nose to the rhinopharynx [1,2]. CA is also characterized by the medialization of the lateral pterygoid plate, which forms the lateral part of this obstruction [3]. Enlargement of the vomer bone is responsible for the obstruction of the medial part. In patients with bilateral CA, the nasopharyngeal airway is completely obstructed on the affected side. In most patients, this obstruction is bony, but it may also be membranous or a combination of both. In its initial phases, CA may manifest as a troubling nasal airway obstruction or rhinorrhea, typical of unilateral CA, although obstruction may be life-threatening [4].

Patients diagnosed with bilateral CA at birth usually present with extreme obstruction, generally manifesting as cyclical cyanosis that is exacerbated when the patient cries [5]. Difficulties passing a suction catheter through a patient’s nasal passages should prompt a thorough diagnostic evaluation of the nasopharyngeal anatomy. Patients can be diagnosed by fiber-optic endoscopy followed, when needed, by computed tomography [6]. A McGovern nipple should be employed to sustain the patient’s respiratory airways and to ease feeding. Surgery is the treatment of choice, with intranasal Hegar’s dilator (IHD) and transnasal endoscopic excision (TNEE) being two of the surgical methods currently used to treat CA. Depending on the severity of the obstruction, surgical intervention may be delayed in patients with unilateral CA until the nasal passages become larger, enhancing the likelihood of successful surgical repair [7].

CA has been estimated to occur in 1 of 5,000-7,000 births, with a 2:1 female predominance [8]. Obstructions are mixed bony-membranous type in 70% of patients and purely bony type in others [9]. CA is unilateral in two-thirds of patients, affecting the right nasal cavity in 71% of those with unilateral CA. The unilateral form may remain undiagnosed for years, whereas bilateral CA, which is generally characterized by severe respiratory distress with intermittent cyanosis exacerbated by crying, is usually diagnosed soon after birth [10]. Although several studies have described the manifestations and management of CA, most of these studies were case series without adequate standardization, making comparisons difficult [2]. Questions remain regarding optimal treatment, including the time of surgery, access to the surgical field, surgical procedure, pharmacological treatment before and after surgery, need for stent implantation, and stent maintenance.

To our knowledge, no randomized clinical trials to date have compared the surgical techniques currently employed to treat and manage CA [11]. Thus, the relative advantages and disadvantages of these procedures have not been definitively determined. Moreover, most studies do not differentiate between patients presenting with unilateral and bilateral CA, with few case series separating these two patient categories [4,6,12].

This study is a retrospective evaluation of the outcomes and effectiveness of IHD and TNEE in patients with CA who were treated at Al-Noor Specialist Hospital, Makkah, Saudi Arabia, over a 20-year period between 1997 and 2017. This study also analyzed the difference in re-stenosis rates in patients who underwent surgery using the two techniques.

## Materials and methods

The medical files of patients who were diagnosed with CA and managed in the Otolaryngology Department of Al-Noor Specialist Hospital between 1997 and 2017 were retrospectively evaluated. CA was classified by tomographic analysis, and patients were treated surgically with TNEE or IHD by trained otolaryngologists. Factors recorded included age at diagnosis, age at surgery, nationality, gender, type of CA (unilateral/bilateral and bony/membranous/mixed), surgical intervention used for initial repair (IHD or TNEE), revision surgery, if necessary, and re-stenosis after both primary and revision. This study was approved by the Institutional Review Board of Al-Noor Specialist Hospital.

Statistical analysis

All statistical analyses were performed with SPSS Statistics for Windows, Version 17.0.2 (SPSS Inc., Chicago, IL, USA). Quantitative parameters were reported as median, 25th and 75th percentiles, and minimum and maximum values. Differences between strata were analyzed using the Mann-Whitney U test. Categorical variables were reported as number and percentage, with differences between groups calculated using the chi-square distribution with Fischer’s exact test.

## Results

**Figure 1:**
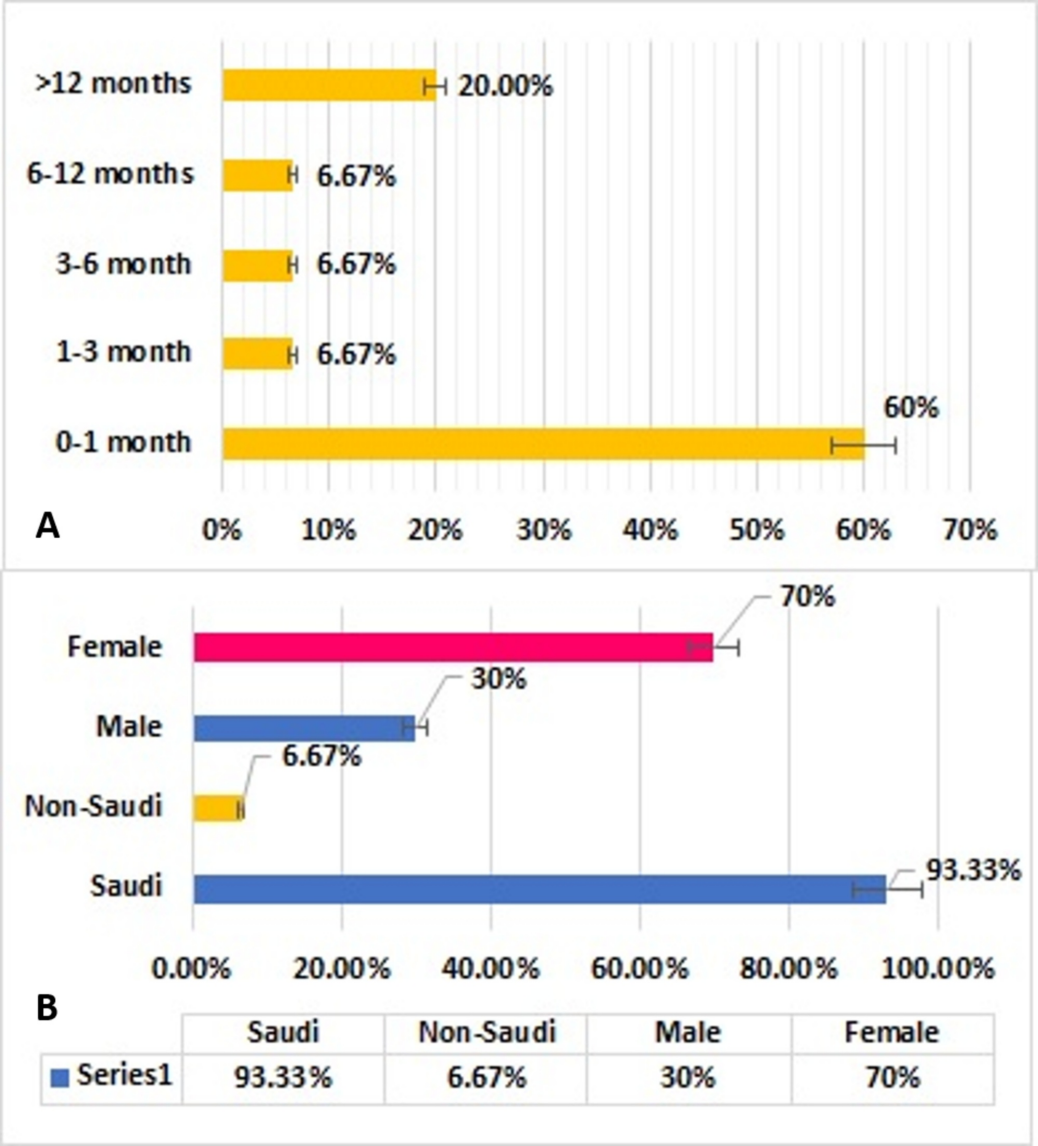
Distribution of patients with choanal atresia by (A) age group at the time of diagnosis and (B) gender and nationality (N=30)

**Figure 2:**
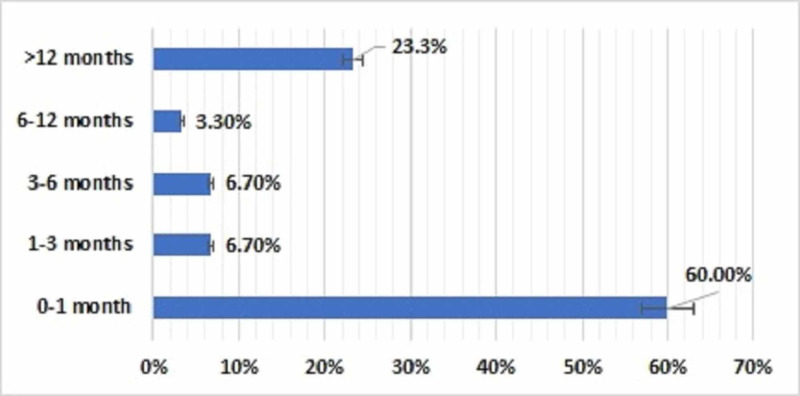
Distribution of patients with choanal atresia by age at the time of surgery (N=30)

This retrospective analysis included 30 patients with CA treated over a 20-year period, including 21 (70%) girls and 9 (30%) boys. Of these patients, 28 (93%) were Saudi nationals and 2 (7%) were of non-Saudi nationals. At diagnosis, 24 (80%) patients were younger than one year, including 18 (60%) younger than one month, 6 patients were aged between one month and one year of age, 2 (6.7%) were between one year and five years of age, and 4 (13%) were older than five years (Figure 1). Of the 30 patients who underwent surgery for CA repair, 18 (60%) were younger than 1 month, two (6.7%) were one to 3 months old and 3 to 6 months old, respectively, one (3.3%) was aged 6 to 12 months, and seven (23.3%) were older than 1 year (Figure 2).

**Figure 3:**
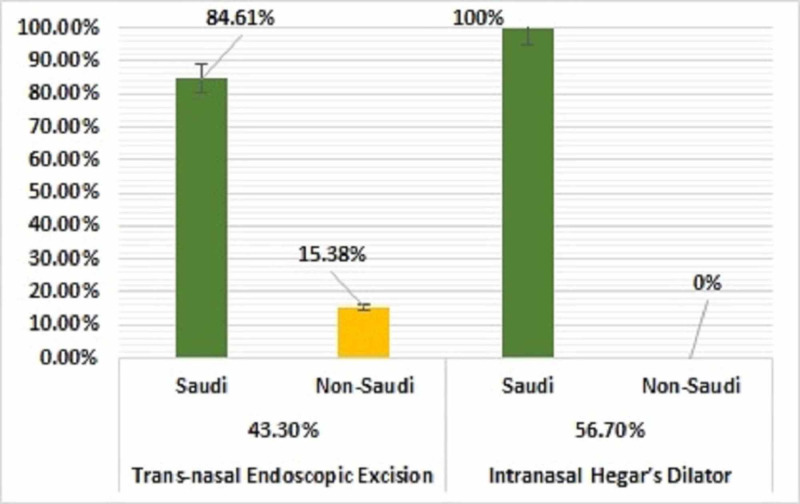
Surgical approaches used to treat patients with choanal atresia and distribution by patient nationality (N=30)

**Figure 4:**
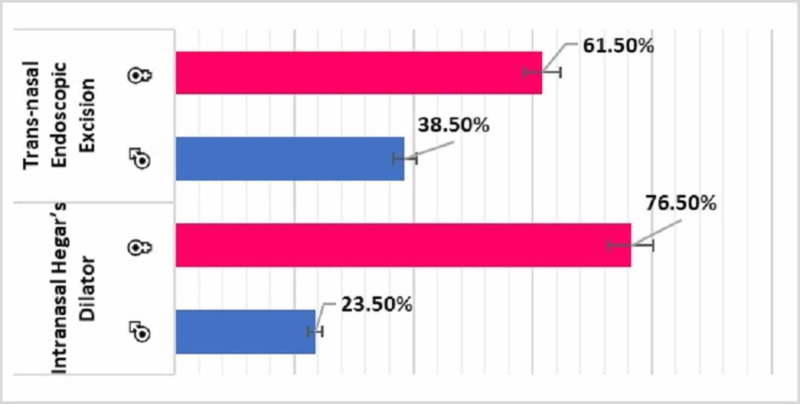
Surgical approaches used to treat patients with choanal atresia and distribution by gender (N=30)

Of the 30 patients, 17 (56.70%) underwent IHD and 13 (43.30%) underwent TNEE for CA repair (Figure 3). All 17 patients who underwent IHD were Saudi nationals, whereas of the 13 patients who underwent TNEE, 11 (84.61%) were Saudi nationals and two (15.38%) were not. The 17 patients who underwent IHD included 13 (76.50%) girls and 4 (23.50%) boys, whereas the 13 patients who underwent TNEE included 8 (61.50%) girls and 5 (38.50%) boys (Figure 4).

**Figure 5:**
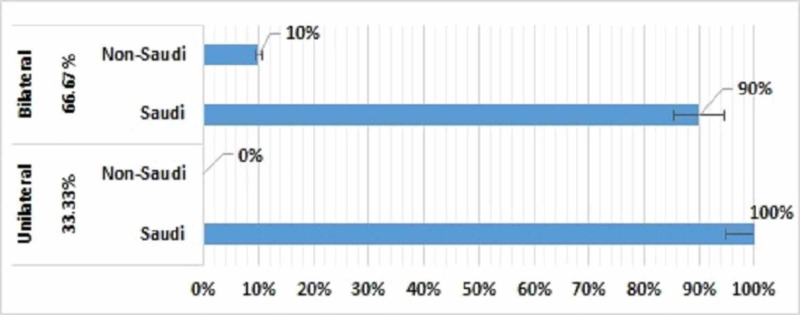
Distribution of patients with choanal atresia by side and nationality (N=30)

**Figure 6:**
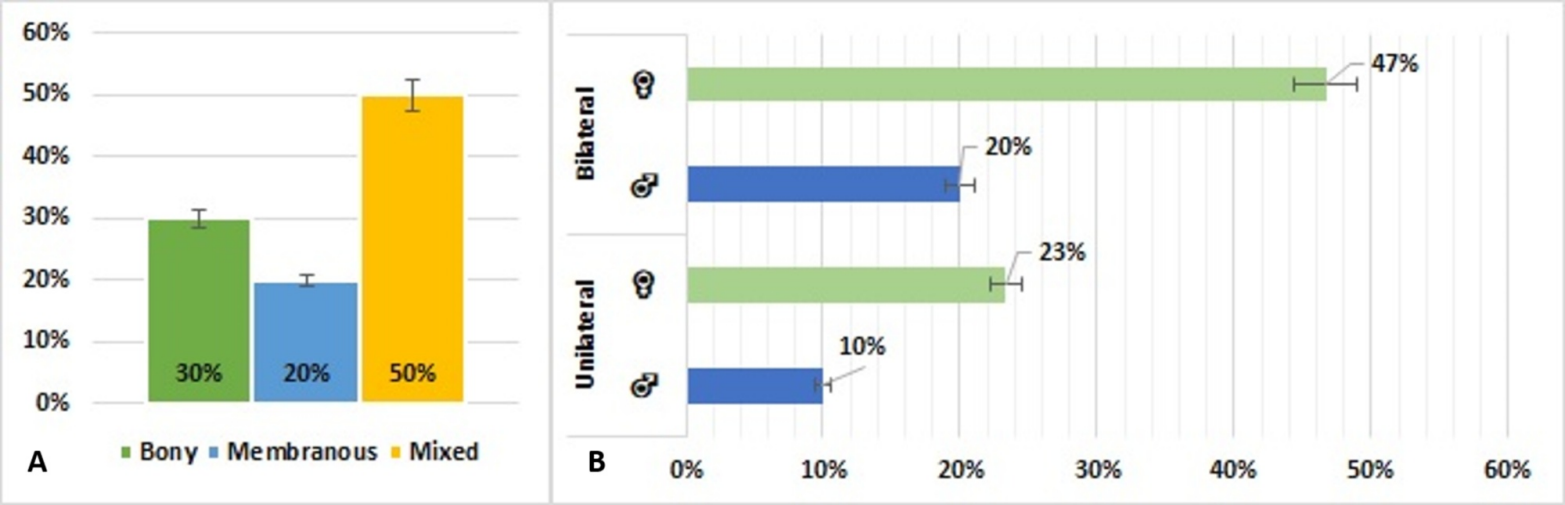
Distribution of patients with choanal atresia by (A) type and (B) side and gender (N=30)

Of the 30 patients, 10 (33%), all Saudi nationals, had unilateral blockage, whereas 20 (67%) patients, 18 Saudi nationals and 2 non-Saudis, had bilateral blockage (Figure 5). The 10 patients with unilateral blockage consisted of 7 girls and 3 boys, whereas the 20 patients with bilateral blockage consisted of 14 girls and 6 boys. Fifteen (50%) patients had mixed CA, nine (30%) had bony CA, and six (20%) had membranous CA (Figure 6).

**Figure 7:**
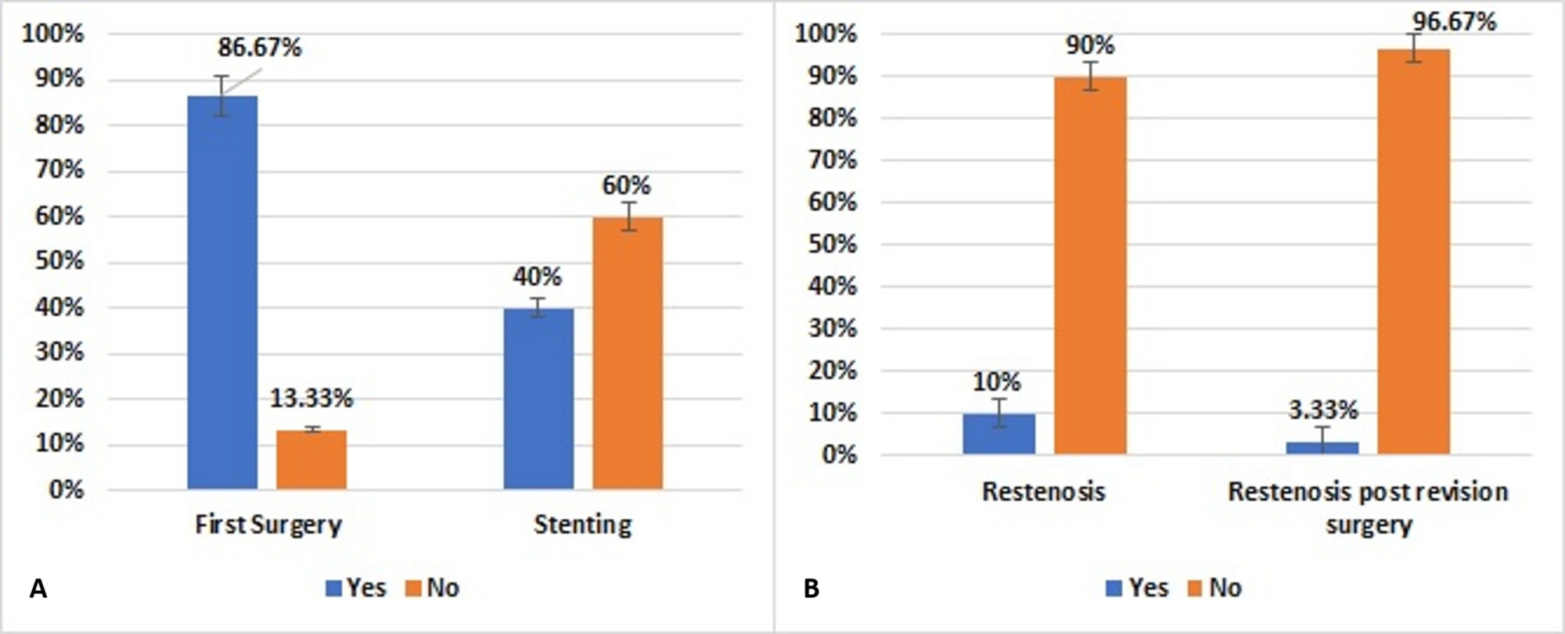
Distribution of patients with choanal atresia who underwent (A) primary surgery and stenting and (B) re-stenosis and re-stenosis post-revision surgery (N=30)

Of the 30 patients, 26 (86.67%) underwent primary surgery for CA, whereas 4 (13.33%) underwent revision surgery. Of the latter four patients, three and one had undergone primary IHD and primary TNEE, respectively. Of the 26 patients who underwent primary surgery, 14 (54%) underwent IHD and 12 (46%) underwent TNEE. Three patients experienced re-stenosis after primary surgery and one after revision surgery (Figure 7). The patient who experienced re-stenosis after revision surgery had undergone IHD for revision surgery. Of the patients who did not experience a second re-stenosis after revision surgery, 55% underwent IHD and 43% underwent TNEE for revision surgery. Twelve (40%) patients underwent stenting, whereas 18 (60%) did not.

None of the differences observed in this study was statistically significant, likely because of the small study sample.

## Discussion

CA is one of the most prevalent major nasal abnormalities worldwide, with an incidence of 1:4000 to 1:10000 live births, and is more common in girls than in boys [13,14]. Similarly, of the 30 patients in this study, 21 (70%) were girls and 9 (30%) were boys. Moreover, 28 (93%) were Saudi nationals, whereas only 2 (7%) were not. Of these 30 patients with CA, 18 (60%) were under one month of age, six (20%) were one to six months of age, two (7%) were one to five years of age, and four (13%) were older than five years. Because CA is a congenital disorder, it was not unexpected that 80% were diagnosed at an age younger than six months.

The optimum age for surgical intervention in patients with CA is unclear, although there is a consensus that surgery should be delayed in patients with unilateral CA and non-serious airway or feeding problems until after one year of age [6,15,16,13,17]. CA repair should be delayed for as long as possible to restrict the enlargement of the surgically created aperture as the surrounding tissues grow and the aperture narrows as the patient matures. Of the 30 patients in our study, 26 (87%) underwent surgery at an age of less than 3 months and almost all at an age of less than 12 months. Early surgery may have been due to the severity of their condition and the seriousness of blockage of breathing and feeding.

The management of osseous/bony CA has been reported to be more challenging than the management of membranous CA, perhaps due to neo-osteogenesis in the former [8,18,19]. Currently, transpalatal and transnasal approaches are the most common surgical repairs for CA. The transpalatal method, however, has several disadvantages including prolonged operation time and the risks of palatal fistula, palatal muscle dysfunction, crossbite, and disturbances in dentoalveolar growth [20]. Our study compared two surgical interventions: IHD and TNEE. The frequency of TNEE was higher than that of IHD, indicating that TNEE was generally preferred. IHD tended to be performed more frequently in boys, whereas TNEE tended to be performed more frequently in girls.

Generally, the incidence of unilateral CA is higher than that of bilateral CA [21]. Our study found that, of the 30 patients with CA, 10 (33%) had unilateral CA, whereas 20 (67%) had bilateral CA. This reversed incidence may have been due to a referral bias in that patients with bilateral CA may be more frequently referred to our high complexity hospital. The 20 patients with bilateral CA included 14 girls and 6 boys, whereas the 10 patients with unilateral CA included 7 girls and 3 boys.

CA can be classified into three types: bony, membranous, and mixed [21]. Of the 30 patients in our study, 15 (50%) had bony-membranous (mixed) type of CA, 9 (30%) had bony type of CA, and 6 (20%) had membranous type of CA. This finding confirmed that CA consists not only of a membrane in the posterior part of the nasal cavity but also a medialization of the pterygoid processes and the entire lateral nasal walls [22].

Of the patients in our study, 26 (87%) patients underwent primary and 4 (13%) underwent a revised CA correction procedure. Only three (10%) patients experienced re-stenosis after surgery. Of the four who underwent revision surgery, three (75%) underwent IHD and one (25%) underwent TNEE for primary CA repair. The rate of second re-stenosis after revision surgery was only 3%, with these patients having undergone IHD for revision surgery. Rates of re-stenosis and revision surgery have been reported higher in patients with syndrome associated than isolated CA [8,18,23-26].

Of the 30 patients in our study, 18 (60%) underwent stent placement to prevent the narrowing of the newly formed lumen while it was still healing. However, stents have been reported to cause severe damage including abrasion of the nasal mucosa, which can lead to granulation tissue, scar formation, microbial overgrowth, and occluded mucous drainage, reducing patency [9,26-27]. Our findings are in good agreement with those several studies reporting no differences in rates of patency between patients who did and did not undergo stenting after surgery [7,9,11,16,26,28]. The difference was not statistically significant, probably due to the limited number of patients. Multicenter studies including larger numbers of patients are therefore needed, as are more prospective trials comparing IHD and TNEE.

## Conclusions

This study found that IHD and TNEE had comparable treatment outcomes in patients with CA treated at a hospital in Saudi Arabia. Rates of re-stenosis and the need for revision surgery were independent of either surgical approach. The limited number of patients in our study suggests a need for additional studies including prospective and multicenter studies that include larger numbers of patients.
